# Gut Failure: A Review of the Pathophysiology and Therapeutic Potentials in the Gut–Heart Axis

**DOI:** 10.3390/jcm12072567

**Published:** 2023-03-29

**Authors:** Dionysis Matsiras, Sofia Bezati, Ioannis Ventoulis, Christos Verras, John Parissis, Effie Polyzogopoulou

**Affiliations:** 1 Emergency Medicine Department, Attikon University Hospital, Rimini 1, 12462 Athens, Greece; 2 Department of Occupational Therapy, University of Western Macedonia, 50200 Ptolemaida, Greece; 3 Emergency Medicine Department, National and Kapodistrian University of Athens, 15772 Athens, Greece

**Keywords:** heart failure, gut, inflammation, intestinal barrier integrity, TMAO, SCFA, microbiota, therapeutic potential

## Abstract

Despite considerable advances in the field, heart failure (HF) still poses a significant disease burden among affected individuals since it continues to cause high morbidity and mortality rates. Inflammation is considered to play a key role in disease progression, but the exact underlying pathophysiological mechanisms involved have not yet been fully elucidated. The gut, as a potential source of inflammation, could feasibly explain the state of low-grade inflammation seen in patients with chronic HF. Several derangements in the composition of the microbiota population, coupled with an imbalance between favorable and harmful metabolites and followed by gut barrier disruption and eventually bacterial translocation, could contribute to cardiac dysfunction and aggravate HF. On the other hand, HF-associated congestion and hypoperfusion alters intestinal function, thereby creating a vicious cycle. Based on this evidence, novel pharmaceutical agents have been developed and their potential therapeutic use has been tested in both animal and human subjects. The ultimate goal in these efforts is to reverse the aforementioned intestinal derangements and block the inflammation cascade. This review summarizes the gut-related causative pathways implicated in HF pathophysiology, as well as the associated therapeutic interventions described in the literature.

## 1. Introduction

**Figure 1:**
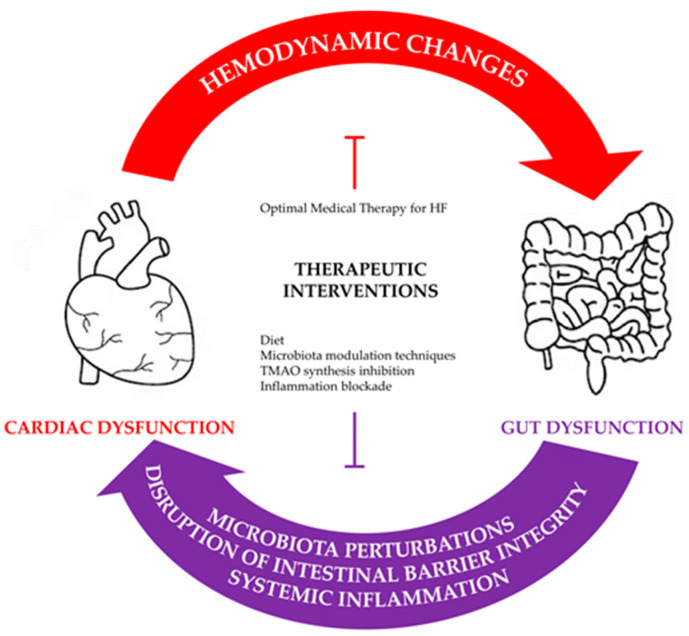
The gut–heart axis and its potential therapeutic targets.

Heart failure (HF) constitutes a global healthcare burden which affects approximately 64,300,000 people worldwide [1]. Despite the significant progress that has been achieved in the fields of prevention, diagnosis and treatment, HF remains a leading cause of cardiovascular morbidity and mortality. It represents a major causative factor of hospitalization among patients over 65 years old and it is associated with a poor prognosis. This is attested by the fact that HF hospitalization per se carries a high risk of poor outcomes, with an estimated 5-year mortality rate of approximately 50% [2,3]. HF is a clinical syndrome, caused by a structural or functional disease, which clinically manifests as lung and/or peripheral congestion and results in decreased cardiac output and inadequate peripheral tissue perfusion [4]. Modern pharmacotherapy targets the neurohormonal axis which encompasses the renin angiotensin aldosterone system and the sympathetic nervous system. However, even in studies characterized by high levels of compliance with optimal medical therapy, it has been shown that mortality remains high, especially after hospitalization [5,6]. Therefore, ongoing research is being performed in pursuit of alternative pathophysiological mechanisms in HF that would expand our understanding of the complexity of the disease and allow us to identify and target new pathways with a view to further improving and individualizing the treatment of patients with HF. As a matter of fact, the presence of low-grade inflammation in patients with chronic HF and the initial hypothesis of a possible disruption of the intestinal barrier [7,8] have drawn researchers’ attention to the gut–heart axis, leading to investigations focusing on the deleterious effects of HF on intestinal physiology/microbiota and vice versa. The present review will attempt to provide a plausible link between gut and heart dysfunction and discuss current evidence regarding the gut-related pathophysiological mechanisms that come into effect in the HF setting. Finally, potential therapeutic interventions will be proposed which target the underlying elements of the complex gut–heart axis and have the potential substantially improve HF management in the future (Figure 1).

## 2. Microbiota

The human gastrointestinal tract (GIT) is colonized by a huge constellation of bacteria, archaea, eukaryotes and viruses. Initial colonization occurs during the processes of delivery and breastfeeding, and involves microorganisms present in the vagina and milk, respectively [9]. The formation process of the microbiota population shows both static and dynamic elements. According to several studies, a variety of factors, other than genetics, can produce prominent variations, such as comorbidities, age, dietary habits, alcohol, smoking, medication, and the environment [10,11,12,13,14]. New technologies have allowed researchers to identify and phylogenetically classify the diverse bacterial population. Among different phyla, *Firmicutes* and *Bacteroides* account for more than 90% of the total intestinal microflora; each is composed of hundreds of species, most of which reside in the large intestine [10]. The microbiota and the host co-evolve in parallel and establish a synergistic relationship via a process known as symbiosis. The host provides nutrients and an anaerobic environment for the microbiota to grow and flourish in, while at the same time the microbiota exerts favorable metabolic and immunomodulatory effects on the host, which are evident at both intestinal and systemic levels. Specifically, microbiota expand the host’s digestive capacity and synthesize metabolites and nutrients, which the host would otherwise be unable to produce. Furthermore, they maintain the intestinal mucosal barrier integrity, prevent the overgrowth of pathogenic species and interact with the innate and adaptive immunity by actively regulating its maturation and function [11]. Notably, their effects are not only confined to the local intestinal environment, but also extend remotely to other organs through the regulation of many systemic activities. 

Indigestible carbohydrates, present in high-fiber diets, are fermented by species of the phylum *Firmicutes*, such as *Roseburia, Eubacterium* and *Faecalibacterium prausnitzi*, to form short-chain fatty acids (SCFAs), namely acetate, propionate, and butyrate [15]. SCFAs serve as energy fuel for several cells since they comprise about 10% of the total daily energy obtained via dietary intake [16]. In particular, butyrate is the main source of energy for the intestinal epithelial cells, while the other SCFAs are absorbed into the portal circulation and are mainly used as energy substrates for the hepatocytes. The remaining SCFAs, mostly acetate, enter the systemic circulation and can actually modify cardiovascular (CV) risk factors by exerting regulatory effects on blood pressure, glucose and lipid homeostasis [17,18]. Moreover, SCFAs may modulate immunological responses by downregulating proinflammatory cytokines and upregulating anti-inflammatory cytokines and T regulator cells (Tregs) [19,20,21] and may also prevent the growth of adhesive pathogenic species [22]. 

**Figure 2:**
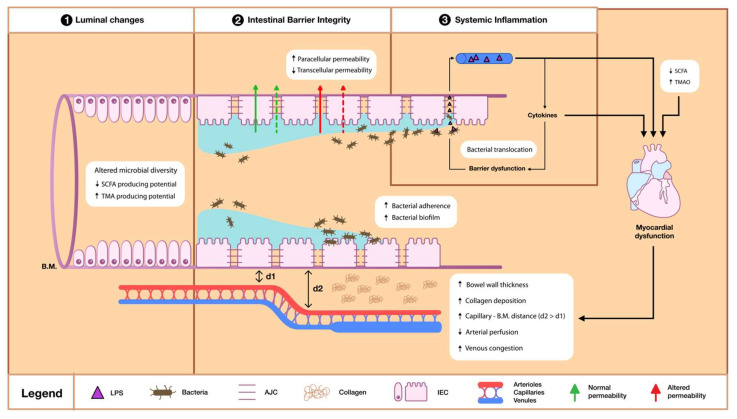
Simplified illustration of the gut-related causative pathways implicated in the pathophysiology of Heart Failure (HF). The underlying pathophysiological mechanisms can be roughly divided into 3 compartments. Within the colonic lumen, altered microbial diversity is characterized by an increase in Trimethylamine (TMA)-producing species and a concurrent reduction in Short-chain fatty acid (SCFA)-producing species, which collectively favor the preponderance of harmful metabolites. Regarding the second compartment, the proper functioning of intestinal enterocytes (IEC) is crucial for maintaining intestinal barrier integrity. IECs receive both luminal and capillary nutritional supply and are physiologically covered by a mucous layer, which restricts their contact with the colonic bacteria. Thus, under normal conditions, they can regulate proper transcellular absorption while at the same time they seal the paracellular pathway via the apical junctional complex (AJC). However, in HF, erosions of the mucous layer and overgrowth of adhesive pathogenic bacteria create the ideal environment for bacteria to impinge on IECs. Moreover, SCFA depletion, in combination with hemodynamic changes (hypoperfusion and congestion) and mechanical bowel wall derangements (collagen deposition), results in decreased trophic status of IECs. These changes disrupt the barrier and lead to an increase in paracellular permeability due to malfunctional AJCs, as well as a concomitant reduction in the transcellular nutrient absorption. In the third stage, bacterial translocation leads to high endotoxin levels in the systemic circulation and recruitment of immune cells with subsequent cytokine release. Successively, cytokines further compromise the already dysfunctional barrier, thus promoting an ongoing bacterial translocation. Cumulatively, systemic inflammation, combined with high Trimethylamine-N-oxide (TMAO) levels and decreased SCFA levels, aggravate myocardial dysfunction. This in turn causes hemodynamic and mechanical changes within the bowel wall, thereby generating a relentless vicious cycle between the dysfunctional compartments of the gut–heart axis. AJC = Apical junctional complex; B.M. = Basement membrane; HF = Heart failure; IEC = Intestinal enterocyte; LPS = Lipopolysaccharide; SCFA = Short-chain fatty acids; TMA = Trimethylamine; TMAO = Trimethylamine-N-oxide.

With regard to the potential contribution of gut dysfunction to HF, the gut–HF hypothesis has been described in the literature [23]. Scientific research has identified possible pathophysiological mechanisms, which are implicated in this untoward interaction and can be classified into categories based on the dysfunctional compartment. According to this classification, the underlying mechanisms may include changes occurring in the intestinal lumen, alterations affecting the intestinal barrier integrity, and inflammatory perturbations involving the systemic circulation (Figure 2).

## 3. Pathophysiological Derangements in the Gut–Heart Axis

### 3.1. Intraluminal Derangements of the Microbiota Composition 

#### 3.1.1. Altered Microbiota Composition and SCFA Levels

Studies into the gut microbiota of patients with HF reveal an altered microbiota composition, characterized by two major population changes: an increase in pathogenic species and a decrease in SCFA-producing species. By applying fecal culture techniques, Pasini et al. reported that patients with chronic heart failure (CHF) had higher levels of Candida, Campylobacter and Shigella species in their stools compared to healthy subjects. This observed increase in pathogenic gut flora was further associated with disease severity, as expressed by New York Heart Association (NYHA) functional classes [24]. Sun et al. applied 16S rRNA gene sequencing to fecal samples of patients with severe CHF and demonstrated that Proteobacteria phylum had substituted the second most physiologically abundant Bacteroides phylum [25]. Additionally, several studies on patients with CHF identified a decreased relative abundance of Ruminococcaceae, Lachnospiraceae [26,27] and Faecalibacterium [28], while another study on patients with acute heart failure (AHF) revealed depletion of Faecalibacterium and reduced abundance of Eubacterium rectale and Dorea Longicatena [29]. Since the latter are known to be organisms which produce butyrate, it becomes evident that the study results are consistent with the presence of a low SCFA-producing capacity in HF states. A loss of species with SCFA-producing capacity occurs regardless of the type of HF, since patients with HF with both preserved ejection fraction (HFpEF) and reduced ejection fraction (HFrEF) develop similar levels of genera depletion [26].

Under normal conditions, the energy requirements of the human heart are mostly covered by the long-chain fatty acids (LCFAs). However, in HF states, the heart generates energy by using SCFAs, ketone and lactate in order to fulfill its metabolic needs. This shift in fuel consumption was observed in a trial comparing nutrient uptake between non-failing and failing hearts, which showed that acetate extraction was increased by 20% in patients with HFrEF [30]. In a similar concept study, it was shown that the SCFA butyrate not only serves as an alternative energy fuel in the failing heart, but also that butyrate oxidation is favored over ketone oxidation for ATP production in both normal and failing hearts [31]. The latter finding actually strengthens the hypothesis that low circulating SCFAs in HF might further exacerbate the disease due to decreased myocyte fuel availability. Metabolic changes are thought to result from a downregulation in the expression of proteins involved in the uptake of LCFA, which is accompanied by an upregulation in the activity of enzymes involved in the oxidation of butyrate [32]. Moreover, SCFA depletion is evident in patients with hypertension, obesity and diabetes mellitus type 2, which collectively constitute risk factors for the development of HF and other CV diseases. Interestingly, in patients with these risk factors, it has been demonstrated that the oral administration of SCFA or a high fiber diet (resulting in increased synthesis of SCFAs) leads to the attenuation of their disease severity [33,34,35]. 

#### 3.1.2. TMAO

Several different taxa are involved in the production of trimethylamine-oxidase (TMAO), which exerts deleterious effects on the host. Red meat and seafood (rich in choline, phosphatidylcholine, betaine and L-carnitine) are metabolized by bacteria, via enzymes called lyases, to trimethylamine (TMA). Thereafter, intestinal TMA is absorbed into the portal circulation and reaches the liver, where the hepatic enzyme flavin monooxygenase-3 (FMO-3) catalyzes its conversion to TMAO [36]. TMAO possesses strong proatherogenic and prothrombotic properties since it induces foam cell aggregation and platelet hyperreactivity. It is associated with thrombotic events (acute myocardial infarction or stroke) [37], vascular inflammation through the activation of the NLRP-3 (nucleotide-binding oligomerization domain-like receptor family pyrin domain-containing 3) inflammasome [38], impaired purinergic-induced intracellular calcium release [39] and mitochondrial dysfunction [40]. Owing to its physiology, it is found in higher levels in omnivorous, westernized diets rather than in vegetarian diets [41], and it is closely related to atherosclerotic risk, coronary artery disease (CAD) and ischemic HF [42]. In patients with CHF, TMAO has been shown to be a predictor of mortality, especially given that patients with higher TMAO plasma levels have a poor 5-year prognosis [23]. The predictive value of TMAO on mortality risk has also been confirmed by Trøseid et al., who further reported that TMAO levels were associated with HF severity, as reflected by NYHA class, while higher levels were found in patients with ischemic HF, followed by stable patients with CAD and non-ischemic HF. In the same study, TMAO levels were also associated with indices of congestion, as evidenced by increased levels of pulmonary capillary wedge pressure and mean pulmonary arterial pressure during right-sided heart catheterization [43]. Concerning the different classifications of HF, Schuett et al. reported that TMAO levels were able to predict mortality in patients with HFrEF, but not in those with HFpEF [44]. 

In patients with AHF, TMAO is a predictor of in-hospital and 1-year mortality, especially when combined with clinical risk algorithms and N-terminal pro-B-type natriuretic peptide (NT-proBNP) [23,45]. Israr et al. expanded the panel of the measured gut metabolites and concluded that L-carnitine and acetyl-L-carnitine were superior in predicting short-term outcomes in patients with AHF, whereas TMAO outperformed carnitine-related metabolites in the prediction of long-term outcomes [46]. However, the correlation between TMAO and adverse outcomes was found to be strongly influenced by ethnicity, with such an association being actually present only in Caucasian populations [47]. It is important to emphasize that TMAO levels are highly linked to westernized diets, even in the absence of established CV disease, as evidenced by a study of healthy subjects in whom red meat diet was associated with higher TMAO levels in comparison to those generated by a white or no meat diet [41]. 

#### 3.1.3. Amino Acids

Amino acids originating from a high protein diet represent another class of biological molecules involved in the gut–heart axis. Tryptophan, by undergoing complex intestinal and hepatic metabolic degradation, gives rise to various metabolites of the kynurenine and the indole pathways [48], which have been evaluated for their contributing role and prognostic value in HF. Regarding the kynurenine pathway, which is the main metabolic pathway of tryptophan and is associated with inflammation [49], it has been demonstrated that healthy subjects with higher plasma kynurenine-to-tryptophan ratios were more prone to developing HF [50]. In patients with HF, higher plasma levels of metabolites of the kynurenine pathway were correlated with decreased functional capacity [51] and worse prognosis in terms of mortality [51,52]. Imazu et al. demonstrated that plasma levels of indoxyl sulfate (IS), a potent uremic toxin of the indole pathway, were elevated in patients with CHF, with mild increases mainly affecting cardiac systolic function and higher IS levels causing both systolic and diastolic dysfunction [53]. Moreover, a positive association between higher IS levels and CV death or HF-associated rehospitalizations was noted in patients with CHF [54]. The results of the latter study were reproduced by Shimazu et al. in a study of 76 patients with dilated cardiomyopathy and mild-to-moderate chronic kidney disease [55].

### 3.2. Intestinal Barrier Dysfunction

In view of the fact that the pathophysiology behind the gut–HF hypothesis suggests the presence of multiple interactions between its different components, the intestinal barrier (IB) could be perceived as the connecting interface between the intraluminal and the systemic changes. The intestinal enterocytes (IECs), together with the apical junction complex (AJC), represent the mechanical barrier that strictly controls molecular absorption. This mechanical barrier is additionally supported by an outer mucus layer rich in mucin and IgA, as well as by the inner lamina propria where cells of the immune system reside [56]. 

#### 3.2.1. Bacterial Derangements

Derangements in the composition of the microbiota population, in conjunction with impaired microcirculation, may compromise IEC function and cause increased permeability, altered absorption and bacterial translocation. Microbiota play important roles in maintaining barrier integrity through the promotion of goblet cell differentiation, mucus synthesis, immune system modulation and SCFA production [57]. The decreased abundancy of SCFA-producing bacteria in patients with HF [26,27,28,29] may alter the luminal nutritional supply and lead to IEC energy depletion. In addition, SCFAs are thought to inhibit the growth of adhesive pathogenic bacteria; hence, their reduction may also contribute to bacterial penetrability [22]. In animal studies, manipulation of the microbiota population and metabolism, induced by dietary fiber deficiency, led to overgrowth of mucin-degrading bacteria, eventually resulting in colonic mucus erosions and increased bacterial penetrability [58]. Similar results have been observed in studies assessing bacterial disequilibrium in human subjects. Mollar et al. noted a negative association between butyrate levels and small intestinal bacterial overgrowth in patients with AHF [59]. Likewise, in another study which included patients with different types of HF, bacterial overgrowth in the small intestine was highly prevalent and could independently predict poor outcomes in all patients with HF across all ranges of ejection fraction (EF). Specifically, small intestinal bacterial overgrowth was linked to increased risk of CV mortality in patients with HFpEF, whereas in patients with HFrEF it was associated with increased risk of HF rehospitalisation [60]. In parallel, large intestinal bacterial overgrowth was confirmed in specimens of patients with CHF who underwent sigmoidoscopy. Mean bacterial density within the mucus and bacterial adherence to the mucosa and bacterial biofilm were all increased in patients with CHF compared to control subjects [61]. 

#### 3.2.2. Permeability and Absorption Derangements

By using orally administered sugar-probe tests and measuring their urinary excretion, Sandek et al. reported the presence of altered intestinal absorption and permeability in patients with CHF [61]. In particular, passive carrier-mediated transport in the small intestine was assessed by D-xylose, with the results demonstrating decreased transcellular absorption across the IEC. On the contrary, increased paracellular permeability in both the small and large intestine was observed, as evidenced by an increase in the permeability index (expressed as the urinary lactulose/mannitol ratio) and in the sucralose excretion, respectively. This finding was consistent with the presence of a compromised AJC function in patients with HF [61]. AJC refers to three distinct intercellular junctions: the tight junction (also called zonula occludens, mainly consisting of occludin and claudin), the adherens junction (known as zonula adherens) and the desmosome, all of which regulate molecular transport through the paracellular route [56]. SCFAs within the intestinal lumen promote AJC integrity by enhancing the mRNA levels of the tight junction proteins [62]. In addition, cytokines, as discussed later, impinge on the AJC structure and function by promoting occludin endocytosis and blocking its normal transport to the lateral IEC membrane [63]. Secondary bile acids (BAs) have also gained attention for being potentially harmful to the IB. The majority of the primary BAs are reabsorbed in the terminal ileum as part of enterohepatic circulation, and approximately 5% enter the colon, where colonic microbes metabolize them into secondary BAs [64]. When present in high concentrations within the colonic lumen, secondary BAs can damage cell membranes and lead to increased paracellular permeability [65]. In the only prospective observational study related to the concentration of BA in patients with CHF, Mayerhofer et al. reported an increased secondary-to-primary BA ratio, a change driven by a reduction in primary BAs. Although an association between increased secondary to primary BA ratio and reduced overall survival was initially detected upon univariate analysis, upon subsequent multivariate analysis this BA pattern was no longer found to be associated with disease severity and mortality [66].

#### 3.2.3. Trophic Changes

IEC status is mainly dependent on its blood supply. The bowel not only requires a rich blood supply, which normally approximates 25% of the cardiac output under resting conditions [67], but also possesses a unique villous capillary structure which renders it susceptible to hypoxic and trophic changes [68]. In order to evaluate these changes, Krack et al. performed intragastric tonometry tests in patients with CHF who underwent low-intensity exercise and reported increased intragastric carbon dioxide pressure, a marker of intestinal mucosal hypoperfusion [69]. In HF states, trophic changes are likely attributed to increased sympathetic vasoconstriction, increased venous congestion or decreased arterial perfusion, and they may deprive IECs from systemic circulation-derived nutrients. Sympathetic hyperactivity, a deleterious feature present in HF pathophysiology, has been shown to lead to vasoconstriction of both precapillary resistance and postcapillary capacitance vessels, ultimately resulting in bowel hypoperfusion [70]. Regarding arterial perfusion, abdominal ultrasonography in patients with HFrEF demonstrated a 30–43% reduction in mean systolic blood flow of the major splanchnic arteries, namely the superior and inferior mesenteric artery as well as the celiac trunk [71]. Likewise, ultrasonography in patients with CHF revealed increased bowel wall thickness, indicative of splanchnic congestion. Interestingly, the grade of the ascending colon thickness correlated with the degree of colon permeability [61]. The role of congestion was also underscored in a clinical study including patients with decompensated HF, in whom the degree of congestion was associated with IB permeability, as denoted by increased systemic endotoxin levels [8]. Moreover, patients with CHF experience morphological changes in the small intestine. These changes are characterized by collagen depositionand compromise enterocytic nutrition and nutritional absorptiond are. Indeed, biopsies collected from the small intestine of 45 patients with ischemic CHF demonstrated high relative collagen content compared to healthy subjects, as well as an increased distance between the IEC basal membrane and the capillary wall, which indicates the presence of a mechanical barrier within the intestinal wall that acts as an impediment and negatively affects absorption. These changes were even more pronounced in patients with advanced HF and cardiac cachexia [72].

### 3.3. Systemic Inflammation

The deleterious consequence of altered gut permeability culminates in the phenomenon of bacterial translocation in the bloodstream. This triggers the release of various endotoxins, which may activate several systemic inflammatory pathways. The hypothesis that inflammation plays a key role in the pathogenesis of HF was first supported by Levine et al., who identified elevated circulating levels of tumor necrosis factor alpha (TNF-a) in patients with CHF [7]. Although various factors have been presumed to generate cytokine production in HF, such as volume overload, endothelial dysfunction and hypoxia, lipopolysaccharide (LPS) has been proposed as the initiating stimulus of the inflammation cascade according to the gut–HF hypothesis [73].

LPS or endotoxin is a recognized pathogen-associated molecular pattern on the surface of Gram-negative bacteria and which may be identified by the CD14 domain of monocytes and macrophages of the innate immune system [74]. Upon intestinal barrier disruption, LPS enters portal and lymphatic circulation and recruits the immune cells through binding to the Toll-like receptor-4 (TLR-4), which subsequently induces the expression of cytokines and cell adhesion proteins via the NF-κB pathway [75,76]. Both in vitro [77,78] and in vivo [79] experimental studies have identified LPS as a potent inducer involved in the release of various proinflammatory cytokines, mainly TNF-a and interleukin-6 (IL-6). TNF may contribute to myocardial dysfunction and HF progression, which is due to the fact that it is implicated in ventricular remodelling, the depression of cardiac contractility, the induction of apoptosis in endothelial cells and myocytes, and the modulation of the enzymes involved in nitric oxide production and cardiac cachexia [80,81]. TNF levels correlate with disease severity [82] and are higher in patients with cardiac cachexia [83]. Notably, the failing myocardium expresses an abundancy of TLR-4, through which TNF-a may exert its action [84]. In addition, LPS may target platelets [85], as well as vascular endothelial cells, and can activate various coagulation system mechanisms [86], subsequently promoting inflammatory pathways. The role of LPS is not restricted to its immunologic effects, as it also seems to influence cardiac performance by causing ventricular depression [87] and electrophysiological dysfunction [88,89]. 

The role of endotoxin was further examined in an experimental study assessing gut permeability in healthy individuals after the intravenous administration of endotoxin. The authors noticed increased gut permeability along with elevated levels of IL-6, IL-10, TNF-a and interferon gamma (INF-γ) [90]. This finding suggested that the main stimulus for the disruption of IB integrity was systemic inflammation induced by LPS [90,91], which essentially triggered a vicious cycle of ongoing inflammation. TNF-a is known to induce AJC dysregulation by causing the internalization of occludin and increased claudin-2 expression, both of which are functional components of a tight junction. Other cytokines involved in the modulation of claudin-2 expression are IL-4, IL-6, IL-9 and IL-13 [63]. 

Anker et al. suggested that the gut could serve as a potential site for microbial invasion, thus acting as a source of inflammation. They reported that patients with elevated levels of soluble CD14 (which is a marker of endotoxin–cell interaction and shedding from the cell membrane) also exhibited increased levels of TNF-a, soluble TNF receptor 1, soluble TNF receptor 2 and intracellular adhesion molecule-1, which indicated pronounced immune activation [92]. Indeed, in an ex vivo model, LPS was found to augment TNF-a concentrations in a dose-dependent manner in patients with both mild and moderate HF [93]. In another study by Niebauer et al., both the concentrations of endotoxin and the levels of several cytokines were higher in edematous patients with CHF compared to patients with stable CHF. More importantly, intensified diuretic treatment resulted in a decrease in endotoxin levels, thus implying that decongestion can lower the grade of inflammation. Paradoxically, cytokine concentrations remained high [8]. In order to investigate the possible source of endotoxin in patients with AHF, Perschel et al. evaluated the levels of endotoxin in the hepatic veins and compared them with measurements in the pulmonary artery and the left ventricle. Endotoxin levels were significantly higher in the hepatic veins compared to the left ventricle or the pulmonary artery, which is consistent with the hypothesis of gut-derived endotoxin translocation into the circulation. Upon discharge, endotoxin levels were significantly lower, while levels of soluble CD14, TNF-a and IL-6 were reduced, although not in a statistically significant way [94]. In the latter two studies, the differentiation between endotoxin and cytokine course was attributed to increased monocyte or macrophage sensitivity to LPS, even after LPS levels had diminished. The persistent hypersensitivity of activated immune cells led to the prolonged production of cytokines and resulted in the lack of immediate cytokine reduction. This notion has been previously supported by Vonhof et al., who observed increased TNF-a concentrations following ex vivo LPS stimulation in patients with acute decompensated HF [95]. Cellular hypersensitivity after LPS stimulation has also been demonstrated in stable patients with CHF; patients in the cohort group had higher TNF-a concentrations both at rest and after exercise compared to te controls [96]. However, the opposite phenomenon, namely endotoxin desensitization in patients with CHF, has also been reported [97,98], suggesting that the mechanisms involved in LPS-induced immune system activation require further elucidation. On these grounds, based on existing paradoxical evidence that lower serum cholesterol correlates with worse prognosis in patients with CHF [99], the endotoxin–lipoprotein hypothesis has been proposed. According to this, serum lipoproteins may regulate LPS activity by serving as a detoxifying buffer that binds with LPS, impeding its biological function consequently [100,101]. 

Furthermore, downregulation of SCFA production leads to the development of a proinflammatory state in HF. As a matter of fact, butyrate is known to attenuate the TNF-a production induced by the LPS through regulation of the NF-κB pathway [102]. At the same time, acetate decreases the release of other proinflammatory cytokines such as IL-6 and IL-17a through the activation of specific G-proteins located in the enterocyte membrane [62]. In addition, by inhibiting histone deacetylase (HDAC), butyrate promotes the production of the anti-inflammatory mediator IL-22 (known for its protective effect on the intestinal epithelium) and increases the levels of T regulatory cells [103].

Gut dysbiosis may also contribute to the inflammatory processes which are involved in cardiac dysfunction and HF progression. Alterations in microbiota metabolites may disrupt immune homeostasis of the gut and induce T-cell overactivation [104]. Immune cell activation and infiltration have been described in the pathogenesis of HF of various etiologies [105]. The phenomenon has been related to left ventricular remodelling and cardiac fibrosis [106]. Moreover, the extent of CD4+ T-cell activation has been proportionally correlated with HF severity [107], potentially through tumor necrosis factor receptor 1 (TNFR1) signalling. In an experimental study where mice were subjected to transverse aortic constriction (TAC), it was demonstrated that cardiac pressure overload resulted in altered composition of the gut microbiota, which was mediated by T-cell activation. In the same study, gut sterilization attenuated T-cell activation and averted cardiac remodelling and dysfunction [108]. 

Finally, primary local intestinal inflammation has also been put forward as a causative agent leading to CV disease. A Danish nationwide cohort study recruiting more than 4.5 million Danish citizens examined the incidence of ischemic heart disease (IHD) in patients diagnosed with inflammatory bowel disease, as compared with healthy subjects, and reported an increased risk of IHD in the former group [109]. Using the same registries, Kristensen et al. observed that patients with inflammatory bowel disease demonstrated an increased risk of hospitalization due to HF, which correlated with disease severity [110]. Nevertheless, a firm association between inflammatory bowel disease and HF incidence is yet to be proven since a recent study investigating HF incidence in various chronic inflammatory diseases did not find an association with inflammatory bowel disease [111]. 

## 4. Therapeutic Potential

Since HF and gut dysfunction interact through various intricate mechanisms, interventions should aim at a holistic and individualized approach. This needs to be based on the etiology of the underlying cardiac and intestinal disorder and should incorporate various therapeutic targets available for modulation.

CV comorbidities should consistently be recognized and treated as part of either primary or secondary prevention. Of particular importance are those linked to atherosclerosis, such as dyslipidemia, diabetes mellitus, hypertension, obesity and metabolic syndrome. These comorbidities can lead to cardiometabolic diseases and cause de novo HF or exacerbate pre-existing HF, especially when combined with smoking, alcohol and physical inactivity [5].

### 4.1. From the Standpoint of the Heart 

Awareness should be raised among physicians regarding the established, yet often under-recognized, association between gut dysfunction and HF. At all times, physicians should provide optimal medical treatment in compliance with the existing HF guideline recommendations [5]. When available, they should utilize imaging methods, combined with biomarker screening tests, to evaluate the presence of subclinical congestion. The use of point-of care ultrasonography may aid in the determination of the hemodynamic status and the optimization of loading conditions. Apart from focused echocardiography and lung scanning [5,112], abdominal ultrasonography could also assist in HF management by identifying increased bowel wall thickness or ascites, which could potentially point towards the need for more intensified diuresis. Regarding the use of biomarkers, natriuretic peptides, the recommended biomarkers for HF diagnosis [5], are known to be secreted in response to increased cardiac filling pressures and volume overload; hence, their values reflect intravascular, but not extravascular, tissue congestion [113,114]. Suzuki et al. conducted a study on stable patients with CHF, investigating the impact of optimal medical treatment on biomarker levels and adverse outcomes. Although B-type natriuretic peptide (BNP) levels significantly decreased in response to optimal medical therapy, as expected, TMAO levels increased. Even more so, patients with higher TMAO levels had worse prognosis in terms of both mortality and rehospitalizations [115]. This fact underlines the need for the investigation of novel biomarkers which closely and invariably correlate with adverse outcomes and degree of congestion, even in the presence of normal BNP levels. To cover this gap, recent evidence supports the use of biologically active adrenomedullin (Bio-ADM), soluble CD146 (cluster of differentiation 146), and cancer antigen 125 (CA125) as markers of extravascular congestion [113].

### 4.2. From the Standpoint of the Gut 

#### 4.2.1. Diet

Even though diet has a major impact on the microbiota population and gut-derived metabolites, most studies on patients with CV disease lack dietary data. However, multiple diets have demonstrated beneficial effects [116]. The use of a high-fiber diet in murine models of hypertension-induced HF led to the growth of acetate-producing bacteria, reduced blood pressure and ameliorated cardiac hypertrophy and fibrosis [117]. Mayerhofer et al. collected information from diet questionnaires distributed to patients with HF who featured changes in their gut microbiota and noted that several characteristics of the dysbiosis observed in patients with HF, including the altered bacterial diversity and the reduction in butyrate-producing bacteria, were associated with a low dietary fiber intake [118]. The effect of specific diets has also been the subject of several human interventional studies. The Mediterranean diet, characterized by the intake of food rich in dietary fiber and unsaturated fatty acids [119], may be beneficial as an adjunctive non-pharmacological therapy in view of the fact that it increases the number of SCFA-producing bacteria and decreases the levels of TMAO and tryptophan–kynurenine pathway metabolites [50,120,121,122]. In healthy subjects, adherence to the Mediterranean diet was associated with reduced incidence of HF [123,124], while, in patients with CHF, it improved left ventricular systolic and diastolic function [125]. DASH (Dietary Approaches to Stop Hypertension) diet is another diet rich in vegetables, fruits and low-fat dairy products, but limited in sugar, red meat, beverages and fat. DASH has been shown to decrease HF incidence by almost 50% and reduce mortality in female patients with HF [126]. Moreover, patients with HF, strictly abiding by DASH diet for 3 months, achieved better performance in 6 min walk tests and had better quality of life when compared with patients with HF who received the standard of care therapy [127].

On the contrary, the Western dietary pattern is linked to high TMAO levels, even in healthy populations [40]. Interestingly, an interventional study evaluated the dietary origins of choline and concluded that egg-derived choline was the only supplement that did not lead to increased TMAO levels [128]. Thus, in anticipation of newer evidence, and taking into account that TMAO has an established deleterious role in HF pathophysiology [41], the overconsumption of its precursors should be discouraged.

#### 4.2.2. Lyases Inhibition

It is known that various strains utilize bacterial enzymes called TMA-lyases, mainly CutC, to regulate the anaerobic metabolism of choline [129]. Multiple in vitro and in vivo animal interventional studies have shown that lyase inhibition led to reduced levels of TMA [130] and subsequently TMAO, while it also resulted in decreased platelet responsiveness and thrombus formation, thereby attenuating atherosclerosis [131]. Roberts et al. developed a potent inhibitor of the gut microbial TMAO pathway which specifically targets genes of the choline utilization (Cut) cluster that encode CutC and CutD, namely the lyases involved in the conversion of choline into TMA. This inhibitory agent of the CutC and CutD bacterial enzymes was administered to mice and resulted in a reduction in plasma TMAO levels for up to 3 days, eventually reversing diet-induced platelet responsiveness and thrombus formation [131]. Additionally, experimental use of iodomethylcholine, a TMA lyase inhibitor, in murine models of HF has also shown promising results. In fact, in a model of pressure overload-induced HF, created by means of transverse aortic constriction (TAC), the administration of iodomethylcholine to mice fed with choline resulted in the improvement of their cardiac function, the attenuation of fibrosis and the abrogation of adverse ventricular remodelling [132].

#### 4.2.3. Microbiota Modification Techniques

Probiotics are defined as ‘alive microorganisms which when administered in adequate amounts confer a health benefit on the host’ [133], whereas prebiotics are defined as ‘non-digestible compounds that, through their metabolization by microorganisms in the gut, modulate composition and/or activity of the gut microbiota, thus conferring a beneficial physiological effect on the host’ [134]. Probiotics have been utilized in experimental and clinical studies in an attempt to modify the human microbiota by upregulating SCFA-producing species and downregulating those producing TMA. In animal models, the administration of probiotics, such as Bifidobacterium breve, Bifidobacterium longum and Lactobacillus plantarum, led to decreased levels of TMAO via gut microbiota remodelling [135,136]. Apart from TMAO level reduction, probiotic supplementation of Lactobacillus Rhimniosus in rats with induced myocardial infarction was able to attenuate the development of heart failure and ventricular wall hypertrophy [137]. Based on these results, two human interventional randomized controlled trials (RCTs) assessed the influence of Saccharomyces boulardii supplementation on patients with HF. However, conflicting results were reported. In the first trial, patients with either HFrEF or HFmEF (heart failure with mildly reduced ejection fraction) were randomized to receive either Saccharomyces boulardii (*S. boulardii*) or a placebo as a daily oral regimen. Results after 3 months of intervention showed that patients in the *S. boulardii* arm exhibited improvement in their LVEF and reduction in their left atrial diameter [138]. However, a larger-scale RCT, the GutHeart trial, found that supplementation with *S. boulardii* had no effect on LVEF, microbiota diversity or measured circulating biomarkers [139]. 

Prebiotics, such as resistant starch, inulin-type fructans and arabinoxylan-oligosaccharides, serve as growth substrates [140] for various species and can be fermented into SCFAs. Studies show that prebiotics stimulate the Roseburia species, which are among the most abundant butyrate producers in the human gut, and Bifidobacteriae [141]. Even though Bifidobacteria are not individually synthesized SCFAs, they ferment carbohydrates into organic acids, which then serve as substrates for butyrate production by other intestinal microbes [142]. In addition, resveratrol, a natural phytoalexin with prebiotic benefits and poor bioavailability, can decrease the production of TMAO, and consequently attenuate its untoward effects, through the reconstitution of intestinal microflora [143].

Additionally, antibiotics have also been used with the intention of modifying the microbiota and its metabolites such as TMAO [144]. Conraads et al. conducted a study on patients with HF with the aim of investigating the role of intestinal decontamination by administering polymyxin B and tobramycin. Antibiotic intervention was able to reduce LPS in the intestine and feces, as well as the intracellular monocyte concentrations of IL-1β, IL-6 and TNF-α [145]. On the other hand, the GutHeart trial reported no benefit in the systolic function of patients with CHF after therapy with rifaximin [139]. Regarding secondary prevention in experimental models, the oral administration of broad-spectrum antibiotics reduced infarct size in rats with induced myocardial infarction [146]. 

#### 4.2.4. Hepatic Flavin Inhibition

As previously mentioned, FMO-3 is the principal hepatic flavin monooxygenase (FMO) responsible for converting TMA into TMAO and represents a therapeutic target aiming at the final step of TMAO synthesis. Its functional modulation has been achieved in both in vitro and in vivo models by using methimazole [147] and dietary indoles from Brussels sprouts [148]. Zhu et al. investigated the effects of FMO3 inhibition in a murine model and found that FMO3 modulation had a direct impact on systemic TMAO levels and its procoagulant effects, mirrored by platelet responsiveness and clot formation [149]. Similarly, in mice with TAC-induced HF, FMO inhibition ameliorated cardiac dysfunction and fibrosis [150]. These findings imply that FMO inhibition could potentially have a similar therapeutic role in humans. However, it should be noted that FMO3 inhibition is accompanied by several side effects that could strongly affect patient compliance, such as hepatic inflammation [151] and secondary trimethyluria, which causes body secretions to have a fishy odour [152].

#### 4.2.5. Inflammation Blockade

The inhibition of the inflammatory cascade has been tested in various levels. Although the anti TNF-a agent known as etanercept has shown promising results in several small-scale studies with patients with HF [153,154], large-scale studies have failed to reproduce these results and have yielded neutral [155] or even negative results [156] with regard to mortality and hospitalization incidence, thus dampening the enthusiasm for the treatment of chronic inflammation in CHF. IL-1 blockade is a more appealing therapeutic target, considering that inhibition of its receptor by anakinra (an IL-1 receptor antagonist) improved the exercise capacity of patients with HFpEF [157]. Anakinra administration in patients with signs of AHF exerted the same effects after a 12-week regimen and additionally showed a tendency towards reduction in death and HF rehospitalization rates at 24 weeks. However, the positive effects on exercise capacity and reduction of morbidity and mortality were not statistically significant in patients with AHF [158]. The impact of the duration of anakinra therapy remains to be clarified in an ongoing study in which patients with HF will receive an extended 24-week regimen [159].

Other agents with immunomodulatory properties have also been recruited in the pursuit of an efficient inflammatory blockade. On these grounds, ursodeoxycholic acid was tested in a double-blind, randomized, placebo-controlled, crossover trial due to its anti-inflammatory properties. The authors reported improved peripheral blood flow and liver function tests, although no benefit was observed in NYHA class, while TNF-a and IL-6 levels remained unchanged or even increased [160]. Recently, in an experimental study of an HF-induced mouse model, estrogen receptor-β agonists were used as immunomodulatory agents in order to antagonize estrogen receptor-α, whose sustained signaling is presumed to be implicated in the pathological activation of CD4+ cells during CHF. Τhis treatment prevented left ventricular dilatation and adverse cardiac remodelling without affecting other immune cells [161]. Last but not least, anti-LPS immunotherapy, achieved by the immunization of patients with various elements of LPS, seems to be a promising therapeutic technique. Until now, studies have either been conducted only in animals or in humans with sepsis; hence, further research into patients with HF is needed in order to investigate the potential benefits of the treatment method [76]. Although there is ongoing research regarding the blockade of various cytokines, mediocre evidence from anti-inflammatory pathway interventions points towards the need for an alternative approach, possibly focused one on targeting earlier stages in the inflammatory cascade or on sealing the IB and restricting endotoxin translocation into systemic circulation. 

Table 1 summarizes the therapeutic interventions employed in experimental and clinical trials which have the targeted specific pathways along the gut–heart axis that are implicated in the pathophysiology of HF. 

## 5. Conclusions

Although scientific research has partially unravelled aspects of the relationship between gut dysfunction and HF, the exact pathophysiological mechanisms and the relative contribution of each culprit is yet to be discovered. Presumably, HF plays the predominant role and can be considered responsible for initiating this interaction. Hemodynamic abnormalities observed in HF, in the form of both congestion and hypoperfusion, may lead to end-organ injury. In this regard, it is intriguing to conceive of the intestinal manifestations as part of a cardio-intestinal syndrome, along the lines of cardiorenal syndrome. However, the notion that intestinal dysfunction may actually precede HF is also appealing. Within this framework, there are sufficient data correlating certain types of diet, which result in elevated TMAO levels with atherosclerosis. Additionally, inflammation, in the context of inflammatory bowel disease, increases the risk of CAD, as well as the HF-associated hospitalization rate. In order to fully comprehend the complex interplay between the gut and the heart and determine the exact degree, level and direction of the interplay between distinct dysfunctional elements of the gut–heart axis, more large-scale human studies are needed. These studies will need to utilize the current knowledge and a combination of diagnostic tests so as to simultaneously assess the luminal, barrier integrity and inflammatory derangements, thus allowing multiple correlations to be made and firmer conclusions to be drawn. Once the exact sequence of the pathophysiological events has been revealed, the relationship between gut dysfunction and HF can be firmly defined. This, in turn, will allow scientists to establish a multifaceted, structured therapeutic approach, instead of proceeding with single-mechanism-based interventions.

## Figures and Tables

**Table 1:** Promising therapeutic targets aiming at gut–heart axis modulation in HF.

Target	Intervention	Effect	Study Type
Diet	MD	Reduced the incidence of HF	Human interventional [120,121]
		Improved LV diastolic filling in CHF	Human interventional [122]
	DASH	Reduced the incidence of HF Reduced mortality in female patients with HF Better quality of life	Human interventional [123]
Microbiota modulation	Probiotics	Attenuated HF development	Animal interventional [133]
		Conflicting results reported regarding LVEF in patients with HF (either improved LVEF or had no effect on LVEF)	Human interventional [134,135]
	Prebiotics	Stimulated SCFA-producing bacteria	Animal interventional [137]
		Decreased TMAO levels	Animal interventional [139]
	Antibiotics	Decreased intestinal concentrations of LPS and cytokines	Human interventional [141]
		No effect on LVEF	Human interventional [135]
TMAO synthesis inhibition	Lyases inhibition	Decreased TMAO levels	Animal interventional [127]
		Improved cardiac function in murine HF models	Animal interventional [128]
	FMO-3 inhibition	Decreased TMAO levels	Animal interventional [145]
		Ameliorated cardiac fibrosis in induced HF models	Animal interventional [146]
Inflammation blockade	Anti-IL-1	Improved exercise capacity in AHF and CHF	Human interventional [153,154]
	ER-β agonists	Ameliorate LV remodelling in HF-induced mouse model	Animal interventional [161]

AHF = Acute heart failure; CHF = Chronic heart failure; DASH = Dietary approaches to stop hypertension; ER = Estrogen receptor; FMO-3 = Flavin mono-oxygenase-3; HF = Heart failure; IL-1 = Interleukin-1; LA = Left atrium; LPS = Lipopolysaccharide; LV = Left ventricle; LVEF = Left ventricular ejection fraction; MD = Mediterranean diet; SCFA = Short-chain fatty acids; TMAO = Trimethylamine-N-oxide.

## Data Availability

No new data was created or analyzed in this study. Data sharing does not apply to this article.
